# Divergent Liver and Kidney Metabolic Responses to Ketogenic, High-Fat, and Sucrose-Enriched Diets in Mice

**DOI:** 10.3390/nu18071141

**Published:** 2026-04-01

**Authors:** Giulia Grillo, Nathalie Vega, Agnieszka Zaczek, Anna Selmi, Stéphanie Chanon, Aurelie Vieille Marchiset, Alessandra Santillo, Aneta Balcerczyk, Maura Strigini, Luciano Pirola

**Affiliations:** 1 INSERM Unit 1060, South Lyon Medical Faculty, Lyon-1 University, 69495 Pierre Benite, France; 2 Department of Environmental, Biological and Pharmaceutical Sciences and Technologies, University of Campania L. Vanvitelli, 81100 Caserta, Italy; 3 Department of Oncobiology and Epigenetics, Faculty of Biology and Environmental Protection, University of Lodz, 90-237 Lodz, Poland; aneta.balcerczyk@biol.uni.lodz.pl; 4 Mines Saint Etienne, Université Jean Monnet Saint-Étienne, INSERM, SAINBIOSE U1059, 42023 Saint-Etienne, France; maura.strigini@univ-st-etienne.fr

**Keywords:** ketogenic diet, liver metabolism, kidney metabolism, lipogenesis, gluconeogenesis, dietary sucrose, AMPK signaling, metabolic adaptations

## Abstract

Background/Objectives: Feeding with a ketogenic diet (KD), nutritionally devoid of carbohydrates, may be metabolically beneficial. The administration of a KD to mice after previous feeding with a high-fat, high-carbohydrate diet (HFD) induced weight loss, ketonemia, and glycemic normalization. Here, to compare organ-specific responses to KD, we analyzed lipogenic and gluconeogenic enzymes and genes in the liver and kidney of mice submitted to KD versus (i) HFD or (ii) a saccharose-enriched diet. Methods: Liver and kidney were from (i) mice fed a HFD followed by an 8-week switch to a chow diet (CD), KD continuation of HFD, and (ii) mice submitted to CD, KD, or a saccharose-enriched diet for 1 week. Protein expression levels were determined by Western blotting, and gene expression by qPCR. Hepatic lipid accumulation was visualized by red oil-O. Results: Switch to a KD led to a simultaneous decrease in lipogenic FASN (Fatty Acid Synthase), ACC (Acetyl-CoenzymeA Carboxylase), and its phosphorylated form (pACC-Ser79) in the liver and kidney. In parallel, we observed increased activating phosphorylation of AMPK, the kinase responsible for ACC phosphorylation. In the liver, but not in the kidney, the gluconeogenic rate-limiting enzyme G6Pase (Glucose 6-phosphatase) was repressed under a KD. The switch to a CD significantly reduced hepatic fat accumulation, while a switch to a KD did not allow a significant reversal of hepatic fat accumulation, suggesting resilience to hepatic fat loss under KD. Comparison of a KD versus saccharose-supplemented diet showed an opposite expression pattern of lipogenic enzymes. Conclusions: Administration of KD after previous HFD induced convergent repression of lipogenic enzymes in the liver and kidney, and specific repression of G6Pase in the liver, suggesting a role for kidney gluconeogenesis during KD. KD versus saccharose-supplemented diet had opposite effects on lipogenesis and glycemic control, but both induced loss of lean body mass.

## 1. Introduction

The ketogenic diet (KD) is a high-fat, very-low-carbohydrate dietary regimen that promotes a systemic metabolic shift from glucose utilization to fatty acid oxidation and hepatic ketogenesis. First introduced in the early 20th century as a therapeutic intervention for epilepsy and diabetes [1], the KD remains an established non-pharmacological treatment for drug-resistant epilepsy and related metabolic disorders, as reflected in contemporary clinical guidelines [2]. Carbohydrate restriction lowers circulating glucose and insulin concentrations, thereby stimulating mitochondrial β-oxidation of fatty acids and hepatic production of ketone bodies, primarily β-hydroxybutyrate (BHB) and acetoacetate, which serve as major oxidative substrates for peripheral tissues [3]. Ketogenesis is governed by mitochondrial 3-hydroxy-3-methylglutaryl-CoA synthase 2 (HMGCS2), the rate-limiting enzyme that condenses acetyl-CoA and acetoacetyl-CoA to form HMG-CoA, subsequently yielding circulating ketone bodies [4].

Beyond their role as alternative fuels, ketone bodies also function as signaling metabolites that modulate transcriptional programs, inflammatory and redox pathways, and epigenetic regulation, including histone β-hydroxybutyrylation, thereby reshaping cellular metabolism and stress responses [5]. These pleiotropic effects have fueled increasing interest in the KD as a strategy to improve metabolic health and resilience [4].

Despite extensive investigation of the KD’s short-term neurological and metabolic benefits, its long-term systemic consequences remain insufficiently characterized. Although prospective studies demonstrate efficacy and general tolerability, prolonged treatment is frequently accompanied by gastrointestinal, nutritional, and metabolic side effects, including constipation, vomiting, and dyslipidemia, as well as other metabolic disturbances reported in clinical KD cohorts and systematic analyses of KDT adverse events [6,7]. In congenital metabolic conditions treated chronically with KD, long-term data on body composition and bone health are limited to small case series and prospective follow-ups; in one study of adults with GLUT-1 deficiency syndrome maintained on KD for >5 years, no major adverse effects on bone mineral content or body composition were observed, although sample sizes were small and broader generalizability is limited [8,9]. Similarly, early application of KD in neonates and infants with genetic epileptic encephalopathies or refractory seizures highlights therapeutic benefit (e.g., seizure reduction) but also a need for careful safety monitoring, with gastrointestinal side effects and other age-related vulnerabilities frequently reported in pediatric cohorts initiating diet therapy within the first months of life [10,11]. Collectively, these data indicate that the long-term effects of KD have been investigated mainly in clinical populations with mandatory indications, whereas evidence in otherwise healthy or metabolically compromised individuals without neurological disease remains scarce.

Concurrently, KD and related carbohydrate-restricted regimens are increasingly explored for broader metabolic indications. Recent clinical studies support short- and medium-term KD interventions as transitional strategies to reduce body weight and ameliorate features of metabolic syndrome [12,13]. Meta-analyses further suggest therapeutic benefit for glycemic control in type 2 diabetes [14,15]. Additional reports propose potential adjuvant roles for KD in neurodegenerative diseases and certain cancers [16]. Nevertheless, emerging evidence cautions that long-term ketogenic feeding may exert adverse effects on hepatic and renal physiology, highlighting the need for careful assessment of organ-specific safety [17].

The liver and kidney are central regulators of systemic substrate homeostasis, coordinating ketogenesis, gluconeogenesis, and lipid metabolism. Yet how these metabolically active organs adapt to sustained ketogenic feeding, and whether their responses diverge from those elicited by high-fat or carbohydrate-enriched diets, remains poorly defined. In previous work, we demonstrated that mice switched from a high-fat diet to a KD exhibited weight loss, improved glycemia, and marked transcriptional remodeling of ketogenic and ketolytic pathways in both liver and kidney, including induction of the rate-limiting ketogenic enzyme HMGCS2 in renal tissue, suggesting a previously underappreciated ketogenic potential of the kidney [18]. However, the signaling mechanisms underlying these adaptations were not fully described, and direct comparisons between KD and carbohydrate-rich diets were lacking.

Here, we ought to systematically characterize hepatic and renal responses to ketogenic feeding relative to alternative dietary paradigms. Using two complementary nutritional protocols, (i) KD following prior high-fat feeding and (ii) KD compared with a 20% saccharose-supplemented diet, we investigated the regulation of lipogenic and lipolytic pathways, gluconeogenesis, and mitogenic signaling in both organs. This approach enabled the identification of both shared and tissue-specific adaptations to ketogenic versus carbohydrate-rich or obesogenic diets and provided some clues on the long-term physiological and safety implications of sustained ketogenic metabolism.

## 2. Materials and Methods

### 2.1. Animal Protocols

*Dietary switch after HFD administration*: 5-week-old male C57Bl6/J mice were obtained from Charles River Laboratories (France). After 1 week of acclimatization, mice were placed on a high-fat diet for 10 weeks and subsequently randomly assigned into one of three groups—continuing on the high-fat diet (HFD group), a chow diet (CD group), or a ketogenic diet (KD group)—for 8 weeks. The macronutrient composition of each diet is presented in Supplementary Table S1.

**Figure 1:**
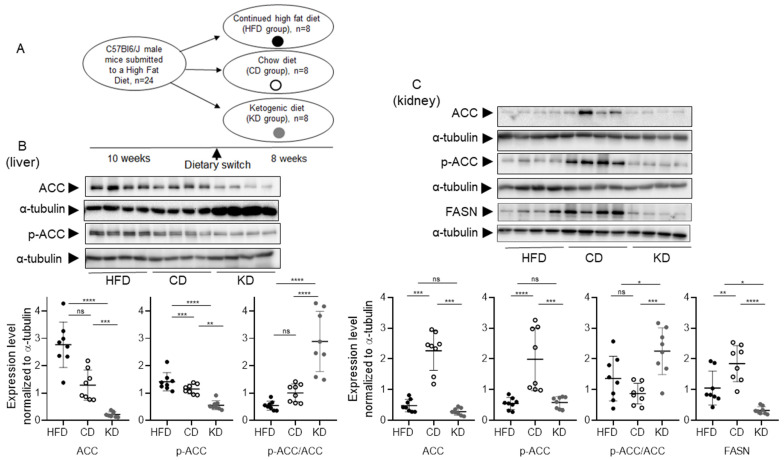
*Effects of HFD, CD, and KD dietary switch on the expression and phosphorylation of lipogenic enzymes ACC2 and FAS in the liver and kidney*. (**A**) Schematic diagram of the nutritional study protocol. (**B**) Upper part: Liver samples were loaded on independent immunoblotting membranes and stained with antibodies directed to ACC and pACC, followed by membrane stripping and re-probing with antibodies directed to a-tubulin. Lower part: relative quantifications of pACC and ACC (normalized to a-tubulin) and of the pACC/ACC ratio. (**C**) Upper part: Kidney samples were loaded on independent immunoblotting membranes and stained with antibodies directed to ACC and pACC and FASN, followed by membrane stripping and re-probing with antibodies directed to a-tubulin. Lower part: relative quantifications of pACC, ACC, and FASN (normalized to α-tubulin) and of the pACC/ACC ratio. (n = 8 biological replicates for each experimental condition). Data were analyzed by one-way ANOVA followed by Tukey’s post hoc test for pairwise comparisons. * *p* < 0.05, ** *p* < 0.01, *** *p* < 0.001, and **** *p* < 0.0001. ns, not significant.

After sacrifice, liver and kidney samples were collected for biochemical analysis. The study protocol is presented schematically in Figure 1A. Metabolic effects of the dietary switch were previously reported, showing weight loss, ketogenesis, and glycemic normalization in mice switched to a KD [18].

*Short-term administration of CD, saccharose-supplemented diet, and KD*: 5 to 10-month old healthy C77B16/J male and female mice fed on chow diet were obtained from the local animal facility (PBES, Platform of Biological Experimental Mice). The mice were randomized into three groups: (i) the first group received a chow diet (CD group, n = 8); (ii) the second group received a chow diet supplemented with 20% saccharose in drinking water (SACCH. group, n = 10); (iii) the third group received a ketogenic diet (KD group, n = 9) for 1 week. Notwithstanding the fact that metabolic responses, as well as liver and kidney physiology, are impacted by the age and endocrine status of aged female mice, we elected to test a KD and 20% saccharose supplementation on both females and males to seek metabolic responses that are robust and independent of sex. Indeed, responses to KD, such as serum BHB elevation and occurrence of β-hydroxybutyrylation, were reproducible in both models. All animal experimental procedures were conducted following institutional guidelines and approved by the French Ministry of Research under study protocol #12127-2017110911058255 v2 valid from 1 January 2021 to 31 December 2026.

### 2.2. Protein Extraction and Western Blotting

To prepare tissue lysates, a lysis buffer containing Tris-HCl, NaCl, KCl, glycerol, sodium-o- vanadate, Nonidet P-40, EDTA, NaF, and protease inhibitors cocktail was added to tissue samples previously ground in liquid nitrogen. After extraction by vortexing and clearance by centrifugation (13,000× *g* for 15 min), supernatants were collected, and the protein levels were determined using bicinchoninic acid (BCA). Lysates were suspended in the Laemmli sample buffer 6× with β-mercaptoethanol. The contents were mixed and boiled at 75 °C for 10 min. The samples were loaded onto 7.5% or 10% SDS-PAGE acrylamide gel. The proteins were transferred to a PVDF membrane (0.2 µm; Amersham^TM^ HYBOND Sigma Aldrich, St Quentin Fallavier, France) followed by blocking with 5% BSA or 5% non-fat milk prepared in Tris-buffered saline with Tween 20 (TBST) for 2 h. The blot was then incubated with a primary antibody overnight, followed by washing, then a 1 h incubation with horseradish peroxidase (HRP)-conjugated secondary antibody. Primary and secondary antibodies used in this study were the following: rabbit monoclonal Acetyl-CoA Carboxylase (C83B10) (Cell signaling #3676); rabbit polyclonal Phospho-Acetyl-CoA Carboxylase (Ser79) Antibody (Cell signaling #3661); rabbit polyclonal Fatty Acid Synthase Antibody (H-300) (Santa Cruz Biotechnology, Heidelberg, Germany, sc-20140); rabbit polyclonal G6Pase-α Antibody (H-60) (Santa Cruz Biotechnology sc-25840); mouse monoclonal anti-tubulin (T5168, Sigma Aldrich, St Quentin Fallavier, France); and secondary antibodies were Anti-Mouse IgG (H+L)-HRP Conjugate and Anti-Rabbit IgG (H+L)-HRP Conjugate (172-1011 and 172-1019, respectively, both from Biorad, Hercules, CA, USA). Standard immunoblotting procedures and ECL detection were used.

### 2.3. Oil Red O Triglyceride Assay

Mouse liver samples were embedded in an optimal cutting temperature (OCT) compound, snap frozen in liquid nitrogen, and stored at −80 °C prior to the mounting of 10 μm cryosections. Liver triglycerides were stained by Oil Red O (ORO-k-250, Biognost, Zagreb, Croatia). Images were analyzed using ImageJ, Version 1.6 and data are expressed as a percentage of the area and the mean lipid droplet size.

### 2.4. RNA Extraction, Reverse Transcription, and Real-Time Quantitative PCR

Total RNA was extracted with the TRI Reagent^®^ (Sigma Aldrich, St Quentin Fallavier, France) in accordance with the manufacturer’s protocol. RNA concentration and purity were determined by measuring absorbance with a NanoDrop 2000 spectrophotometer (Thermo Fisher Scientific, Illkirch, France). cDNA synthesis was performed using the PrimeScript™ RT Reagent Kit (Takara, Saint-Germain-en-Laye, France), starting from 1 μg of total RNA in a total reaction volume of 20 μL. Quantitative real-time PCR (qPCR) amplification was performed using a Rotor-Gene Real-Time PCR System (Quiagen, Courtaboeuf, France). Amplification reactions contained 5 μL of cDNA, 5 pmol of forward and reverse primers, and 15 μL of ABsoluteTM QPCR SYBR Green Mix (ABgene, Illkirch, France). The sequences of the primers used are reported in Supplementary Table S2. As a quality control, qPCR amplicons were analyzed by agarose gel. Analyzed genes were normalized against the β-actin gene.

### 2.5. Quantitative Magnetic Resonance (QMR) Measurements

QMR, allowing for the determination of mice lean mass, fat mass, and liquid mass, was performed on a Time Domain NMR Bruker Minispec Plus apparatus (www.bruker.com, Bandol, France), as per manufacturer instructions. Briefly, mice were placed within the cylindrical plastic holder and then inserted into the acquisition section of the Minispec apparatus.

### 2.6. Statistical Analysis

The results are expressed as mean ± standard deviation (SD). One-way ANOVA, followed by a Tukey post hoc test for pairwise comparisons, was used to compare the differences between experimental groups. Statistical significance was set at *p* < 0.05. Data are presented as mean ± SD. Statistical significances are reported throughout the study as follows: * *p* < 0.05, ** *p* < 0.01, *** *p* < 0.001, and **** *p* < 0.0001.

## 3. Results

### 3.1. A Dietary Switch to a KD Blunts Lipogenesis in Both the Liver and Kidney

C57Bl6/J male mice were fed for 10 weeks with an HFD prior to a switch to a CD or KD, or continued HFD for 8 weeks (Figure 1A). To evaluate the impact of the three diets on the lipogenic potential of the liver and kidney, we assessed the expression and phosphorylation levels of ACC as well as the expression of FASN. Total protein expression of ACC was significantly increased in the liver (Figure 1B) and kidney (Figure 1C) of mice switched to a CD (*p* < 0.0001 as compared to HFD and KD), as it was for FASN in the kidney (*p* < 0.01 vs. HFD, *p* < 0.0001 vs. KD) Figure 1C) and, as previously published [18], in the liver.

ACC, in its activated form, is dephosphorylated [19]. We observed, in the CD group, in parallel to ACC overexpression, significantly higher phospho-ACC levels. However, the calculation of the pACC/total ACC ratio demonstrated a significantly higher ratio in the KD group, both in the liver and kidney. Hence, administration of a KD both suppresses ACC expression and, concomitantly, increases its inhibitory phosphorylation level, suggesting a minimal occurrence of lipogenesis in both the liver and kidney (Figure 1), and a similar regulation of the lipogenic pathway in the two organs.

### 3.2. Under a KD Condition, AMPK Phosphorylation Correlates with ACC Inhibitory Phosphorylation

**Figure 2:**
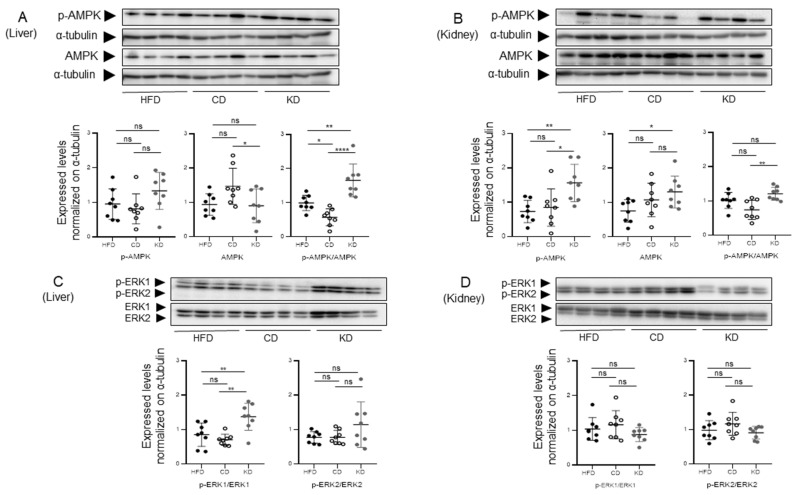
*Effects of HFD, CD, and KD dietary switch on the expression and phosphorylation of AMPK and MAPK signaling enzymes in liver and kidney*. Liver samples (**A**) and kidney samples (**B**) were loaded on independent immunoblotting membranes and stained with antibodies directed to pAMPK and total AMPK, followed by membrane stripping and re-probing with antibodies directed to a-tubulin. Lower part: relative quantifications of pAMPK and total AMPK (normalized to a-tubulin) and of the pAMPK/AMPK ratio. Liver samples (**C**) and kidney samples (**D**) were loaded on independent immunoblotting membranes and stained with antibodies directed to pErk1,2 and total Erk1,2, followed by membrane stripping and re-probing with antibodies directed to a-tubulin. Lower part: relative quantifications of pErk1,2 and total Erk1,2 (normalized to a-tubulin) and of the pErk1,2/Erk1,2 ratio. (n = eight biological replicates for each experimental condition). Data were analyzed by one-way ANOVA followed by Tukey’s post hoc test for pairwise comparisons. * *p* < 0.05, ** *p* < 0.01, and **** *p* < 0.0001. ns, not significant.

To investigate the possible signaling events leading to diet-induced ACC phosphorylation, we monitored the phosphorylation state of AMPK, which classically occurs in a low-energy state [20], and of the mitogen-activated protein kinase isoforms Erk1/2, occurring upon insulin receptor stimulation. Mice switched to a KD exhibited a higher pAMPK/total AMPK ratio as compared to mice switched to a CD, both in the liver and kidney (Figure 2A and B, respectively). In the liver, the pAMPK/total AMPK ratio of K-fed mice was also significantly higher than in mice kept on a HFD. Parallel monitoring of ERK1,2 MAPK total expression and phosphorylation status did not reveal major modulations induced by any of the three diets, neither in liver nor in kidney (Figure 2C and D, respectively). As AMPK mediates the phosphorylation-dependent inactivation of ACC [21], our observation supports the role of an AMPK-ACC pathway, but not MAPK (Figure 2C,D) or PKB/AKT [18], to inhibit lipogenesis in mice switched to a KD.

### 3.3. Changes in Liver Fat Accumulation upon Dietary Switch to CD or KD

**Figure 3:**
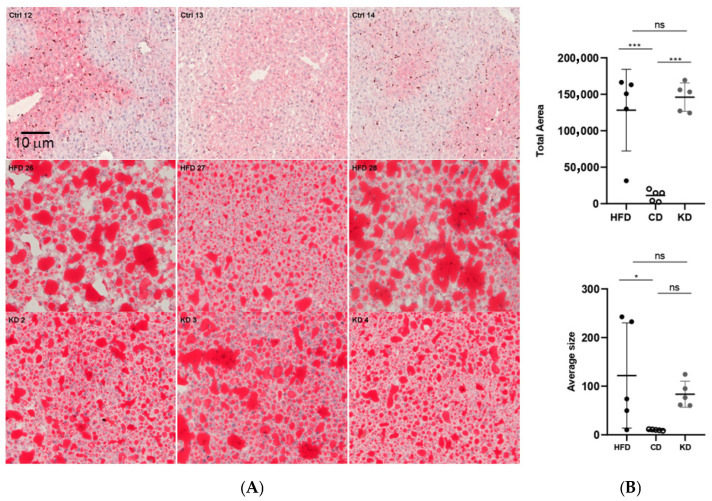
*Effects of HFD, CD, and KD dietary switch on lipid accumulation in the liver*. (**A**) Liver sections stained with Oil RED O from mice switched to a CD (**upper panels**), HFD (**central panels**), and KD (**lower panels**). Scale bar (10 μm) applies to all micrographs. (**B**) Quantifications of the Red OIL O signals expressed as total droplets area (**upper graph**) and droplets average size (**lower graph**). (n = five biological replicates for each experimental condition). Data were analyzed by one-way ANOVA followed by Tukey’s post hoc test for pairwise comparisons. * *p* < 0.05, *** *p* < 0.001. ns, not significant.

Hepatic lipid accumulation is known to occur following a 16-week hypercaloric regimen in mice [22]. To visualize hepatic lipid accumulation, cryosections of livers from mice switched to a KD, CD, or maintained on an HFD were stained with RedOilO. Mice maintained on an HFD displayed extensive lipid accumulation, while mice switched to a CD had a significant reversion of lipid accumulation (*p* < 0.001 CD vs. HFD). Switching to a KD for 8 weeks, in spite of weight loss and glycemic normalization [18] did not lead to a significant loss of hepatic lipids (*p* = n.s. KD vs. HFD, Figure 3, right graphs). Overall, our data argue against loss of HFD-induced hepatic lipid accumulation upon switch to a KD, although some decrease in lipid droplet size was observed, making the lipid droplet size of KD-fed mice not significantly larger than that of mice switched to a CD (Figure 3, left panels).

### 3.4. Potential Hepatic, but Not Renal, Gluconeogenic Repression by KD

**Figure 4:**
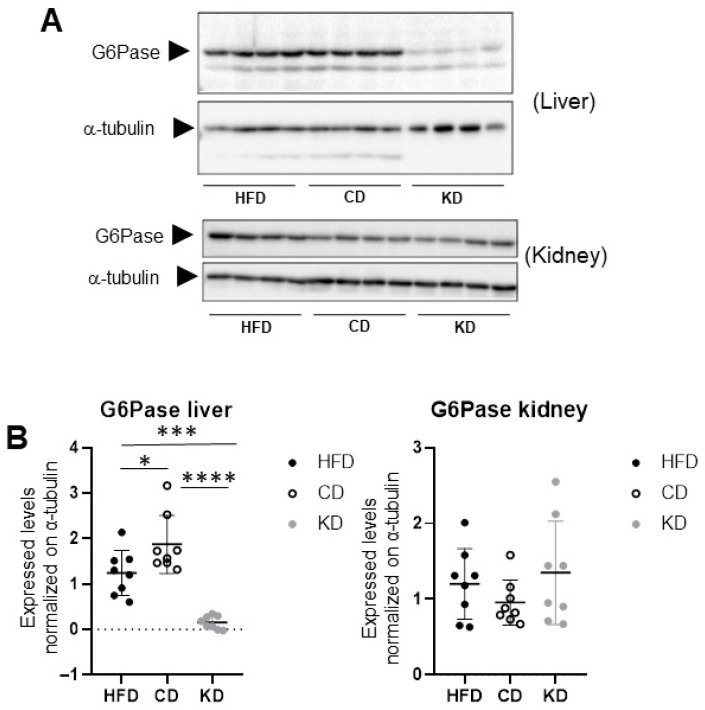
*Effects of HFD, CD, and KD dietary switch on the expression of the gluconeogenic rate-limiting enzyme G6Pase in liver and kidney*. Liver samples and kidney samples (**A**) were loaded on independent immunoblotting membranes and stained with antibodies directed to G6Pase, followed by membrane stripping and re-probing with antibodies directed to a-tubulin. (**B**) Relative quantifications of G6Pase (normalized to α-tubulin) in liver samples (**left graph**) and kidney samples (**right graph**). (n = eight biological replicates for each experimental condition). Data were analyzed by one-way ANOVA followed by Tukey’s post hoc test for pairwise comparisons. * *p* < 0.05, *** *p* < 0.001, and **** *p* < 0.0001.

The protein expression of the rate-limiting gluconeogenic enzyme Glucose-6-phosphatase (G6Pase, [11] was measured in liver (Figure 4A, upper blots) and kidney (Figure 4A, lower blots) protein lysates from mice switched to a KD, CD, or maintained on a HFD. Administration of a KD blunted hepatic G6Pase protein expression (*p* < 0.0001 KD vs. CD, *p* < 0.001 KD vs. HFD) while kidney G6Pase levels remained unaltered (Figure 4B).

### 3.5. Short-Term KD and Saccharose Supplementation Exert Opposite Effects on Ketogenesis and Lipogenesis

**Figure 5:**
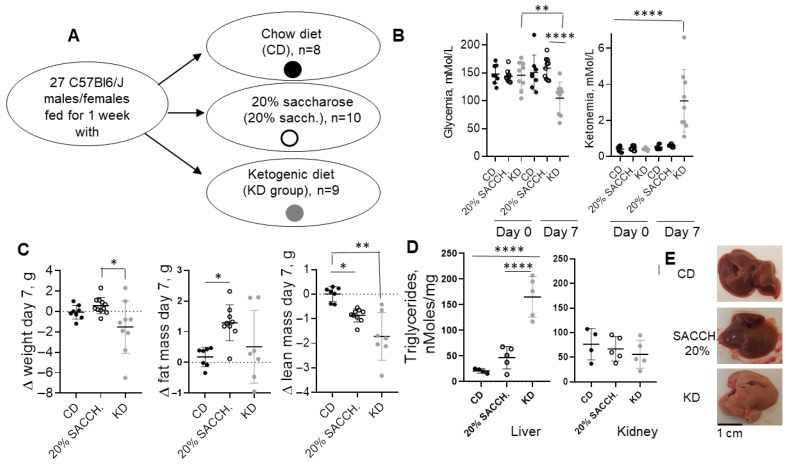
*Metabolic features of a short-term KD versus saccharose-supplemented diet*. (**A**) Schematic diagram of the nutritional study protocol. (**B**) Random (i.e., in fed animals) glycemic values (**left**) and ketonemia (**right**, *p* < 0.01, **, *p* < 0.0001, ****, of KD at day 7 versus all other conditions) at the study onset (day 0) and study end (day 7). *p* < 0.01 (**C**) Change in body weight (**left**), fat mass (**center**), and lean mass (**right**) as compared to baseline measured by QMR. (**D**) Liver triglyceride content. (**E**) Representative pictures of livers at explant. Scale bar (1 cm) applies to all pictures. Statistics: One-way ANOVA with *post hoc* Tukey’s test was used to compare the three diets. * *p* < 0.05, ** *p* < 0.01 and **** *p* < 0.0001.

**Figure 6:**
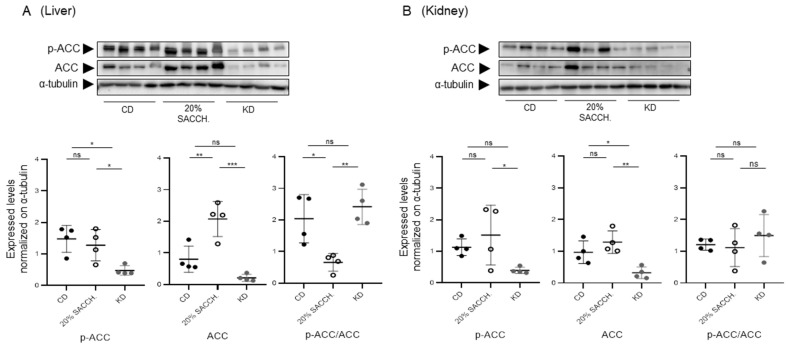
*Opposed effects of a saccharose-rich diet versus KD on the expression and phosphorylation of lipogenic enzymes ACC2 and FAS in the liver and kidney*. (**A**) Upper part: Liver samples were loaded on independent immunoblotting membranes and stained with antibodies directed to ACC and pACC, followed by membrane stripping and re-probing with antibodies directed to a-tubulin. Lower part: relative quantifications of pACC and ACC (normalized to α-tubulin) and of the pACC/ACC ratio. (**B**) Upper part: Kidney samples were loaded on independent immunoblotting membranes and stained with antibodies directed to ACC and pACC and FASN, followed by membrane stripping and re-probing with antibodies directed to a-tubulin. Lower part: relative quantifications of pACC, ACC, and FASN (normalized to α-tubulin) and of the pACC/ACC ratio. (n = 4 for each experimental condition). Data were analyzed by one-way ANOVA followed by Tukey’s post hoc test for pairwise comparisons. * *p* < 0.05, ** *p* < 0.01, *** *p* < 0.001. ns, not significant.

**Figure 7:**
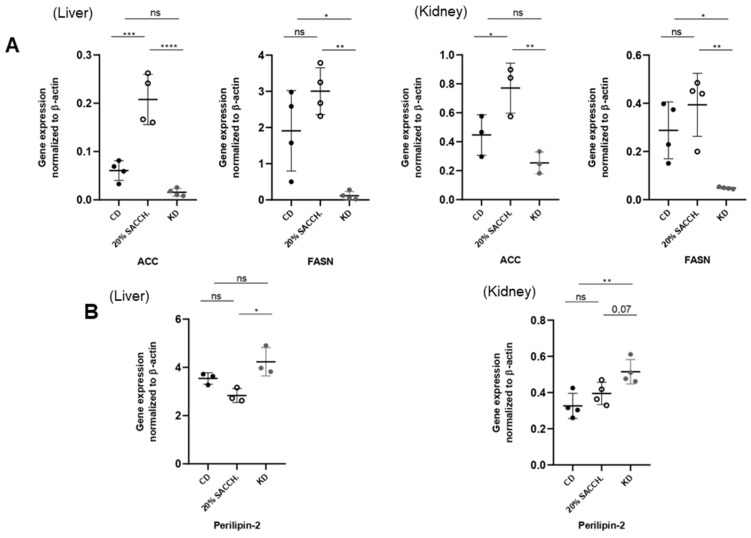
*Opposed effects of a saccharose-rich diet versus KD on the gene expression of lipogenic and lipolytic genes*. (**A**) Left: Gene expression in the liver (**left**) and kidney (**right**) of lipogenic *Acc* and *Fasn*. (**B**) Gene expression in the liver (**left**) and kidney (**right**) of the lipogenic gene perilipin-2. (n = 4 for each experimental condition). Data were analyzed by one-way ANOVA followed by Tukey’s post hoc test for pairwise comparisons. * *p* < 0.05, ** *p* < 0.01, *** *p* < 0.001, and **** *p* < 0.0001. ns, not significant.

**Figure 8:**
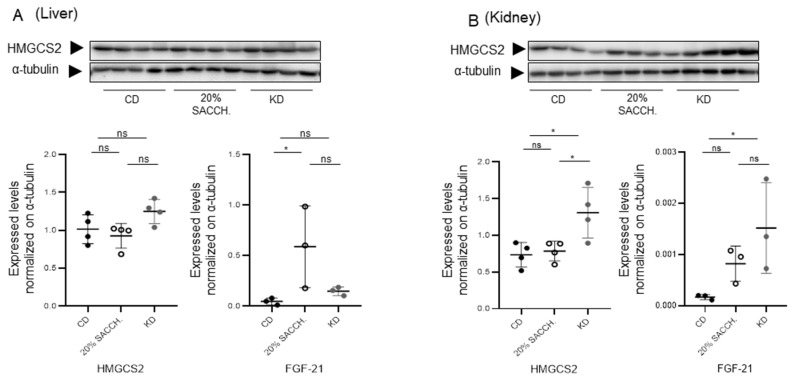
*Hepatic and renal expression of ketogenic diet mediators* HMGCS2 *and Fgf21 in mice on KD and 20% Saccharose supplementation*. Western blot analysis of HMGCS2 in liver (**A**) and kidney (**B**) total protein extracts. Immunodetection of α-tubulin was used as a loading control. Lower graph: quantification of HMGCS2 protein expression and *Fgf21* gene expression in liver (**left** graphs) and kidney (**right** graphs). Protein HMGCS2 quantification was normalized to the α-tubulin signal, and *Fgf21* gene expression was normalized to gene expression of the β-actin gene. Statistics: ANOVA with Tukey’s post hoc test. * *p* < 0.05. ns, not significant.

To evaluate the short-term metabolic effects of a KD, female and male mice (age range 5 to 10 months old) were fed for 1 week with a CD, KD, or a CD supplemented with 20% saccharose administered in drinking water—the latter intervention to model a carbohydrate-rich diet opposite to the carbohydrate-deprived KD (Figure 5A). Fed glycemia and ketonemia were measured in the morning in fed animals at day 0 and at the end of the 1-week period, showing significantly decreased glycemia in the KD group as compared to the saccharose-supplemented group and induction of ketonemia in the KD group (Figure 5B). Over the 7-day protocol, mice’s body weight and fat mass, as measured by QMR, were not altered. However, both the 20% saccharose-supplemented group and, to a greater extent, the KD group displayed significantly decreased body lean mass (Figure 5C). The KD-fed group also accumulated triglycerides in the liver (Figure 5D), as also shown by a clearly defined steatotic appearance (Figure 5E). Administration of 20% saccharose significantly favored hepatic lipogenesis, as shown by increased ACC expression and simultaneous reduction in its inhibitory phosphorylation (Figure 6A), and, contrarily, lipogenesis was blunted under KD (Figure 6A). In the kidney, only a minor and non-significant modulation of lipogenic proteins occurred (Figure 6B). Hepatic gene expression of lipogenic *Acc* and *Fasn* was significantly opposite in both liver and kidney as compared to the 20 saccharose versus KD, and, similarly, lipolytic gene perilipin 2 (*Plin2*) was significantly higher in the KD as compared to the 20% saccharose diet (Figure 7). Finally, expression of the rate-limiting ketogenic enzyme HMGCS2 was mildly induced in the liver and strongly induced in the kidney after KD administration, while *Fgf21* was preferentially induced by the saccharose diet in the liver and by the KD in the kidney (Figure 8).

### 3.6. Induction of Histone Beta-Hydroxybutyrylation by Short-Term KD in Both the Liver and Kidney

**Figure 9:**
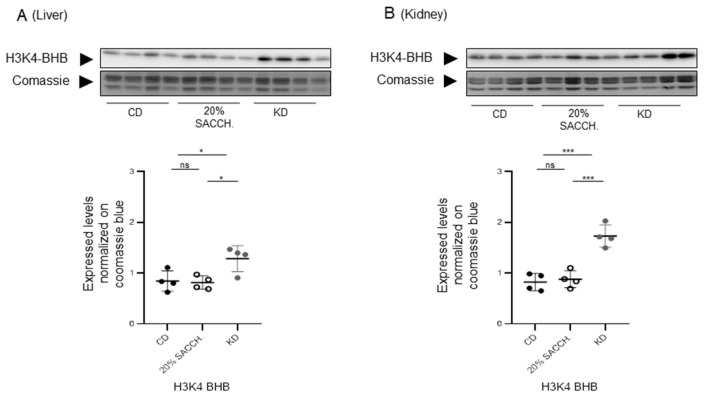
*Effects of 1-week 20% saccharose supplementation and KD on histone β-hydroxybutyrlation*. Acid-extracted histones from liver (**A**) and kidney (**B**) were immunoblotted with antibodies to β-hydroxybutyrylated histone H3 lysine 4 (H3K4–BHB, left graphs). Loading was assessed by Coomassie blue staining. Immunoblotting signal quantification, shown on the bottom, is relative to the Coomassie blue signal. All pairwise statistically significant differences by one-way ANOVA and Tukey’s post hoc test are shown. * *p* < 0.05, *** *p* < 0.001. ns, not significant.

The occurrence of histone β-hydroxybutyrylation was initially discovered in cells treated with BHB and in the liver of starved STZ-treated diabetic mice [23] and in mice submitted to a KD for a prolonged period, i.e., 8 weeks [18]. Here, we evaluated the occurrence of β-hydroxybutyrylation on histone H3 lysine 4 (H3K4–BHB) in the liver and kidney upon a substantially shorter administration of a KD (7 days). H3K4–BHB increased in the liver (Figure 9, left panel) and kidney (Figure 9, right panel). Overall, increased histone β-hydroxybutyrylation in the KD group is a specific chromatin change that we now show takes place on a KD feeding protocol as short as 1 week.

## 4. Discussion

Obesity prevalence worldwide is constantly increasing, while elevated BMI should not be considered per se as a diagnostic criterion for morbidity [24], elevated BMI predisposes to co-morbidities, including type 2 diabetes, cardiovascular conditions, and fatty liver disease. While genetics plays a causal role in monogenic forms of obesity [25], most of the obesity incidence carries a more complex contribution from polygenic scores, in which defined gene variants contribute towards, or against, body weight accrual [26]. Given the impossibility of overcoming the genetic component during the development of an obese state, innumerable studies have addressed surgical, pharmacological, and dietary lifestyle interventions to attain weight loss. While the use of GLP-1 receptor agonists [27] and bariatric surgery [28] is nowadays on the rise, dietary approaches have also received intense scrutiny, as less invasive and perhaps more actionable interventions over the long period, especially for the control of non-morbid obesity [29]. Among others, ketogenic diets have been studied as a nutritional approach for weight loss both in preclinical rodent models [30] and in humans [31]. A dietary carbohydrate intake of <50 g/day [32], typical of ketogenic diets, induces a switch to fatty acid beta-oxidation as a major energy source, concurrent with hepatic synthesis of ketone bodies as an alternative fuel from beta-oxidation-derived AcCoA. Somehow paradoxically, the energy-dense ketogenic diet has a metabolic convergence with fasting or caloric restriction, since these nutritional states rely on ketogenesis. A span of studies in rodents demonstrated that a ketogenic diet prolongs lifespan and health span [33], improves memory [34], and ameliorates metabolism [35], although the long-term consequences of such dietary intervention may be metabolically detrimental [36]. Conversely, dietary overload of carbohydrates has also been proposed to be noxious, raising the concept of “carbotoxicity” [37]. As an example, consumption of sugar-sweetened beverages (SSBs) is linked to a significant contribution towards Type 2 diabetes and cardiovascular disease worldwide [38].

Excess carbohydrate intake is funneled towards energy storage through lipogenesis [39]. Additionally to the liver, the main lipogenic organ, the kidney is also capable of de novo lipogenesis, which is the process of synthesizing new fatty acids from non-lipid precursors, such as glucose and amino acids [40,41].

During ketosis, the body switches from glucose metabolism to fat metabolism to produce energy. As a result, there is a decrease in the availability of glucose and an increase in the levels of ketone bodies in the bloodstream. This leads to attenuated expression and activity of enzymes involved in lipogenesis in various tissues, including the kidney, in which lipogenesis has been recently shown to take place [42].

In our study, we analyzed the differential and organ-specific responses of the liver and kidney, in two different nutritional protocols: (i) prior HFD followed by a switch to KD and (ii) short-term KD versus saccharose supplementation. In HFD-fed mice, switch to a KD induced a significant repression of lipogenic proteins and genes in both liver and kidney, as demonstrated by reduced protein expression levels of ACC and FASN coupled with increased inhibitory phosphorylation of ACC, which represents a modification known to block fatty acid biosynthesis (Figure 1 and Figure 2). The inhibition of the lipogenic pathway in both organs was further confirmed by the increased activating phosphorylation of AMPK, the energy sensor that downregulates anabolic lipid synthesis in states of carbohydrate deprivation. The results of repression of lipogenesis through the AMPK–ACC–FASN axis provide evidence that a ketogenic condition induces a systemic shift away from de novo fatty acid synthesis toward enhanced fatty acid oxidation, thereby limiting further lipid deposition in metabolically active organs [30]. Of note, these findings extend previous studies by demonstrating that this metabolic reprogramming is not limited to the liver, the main center of lipid metabolism, but is also occurring in the kidney. Recently, it was reported that the kidney possesses the enzymatic machinery for de novo lipogenesis and can modulate lipid fluxes in response to nutritional cues, supporting its emerging role in systemic energy homeostasis [42].

While similarities were observed in terms of lipogenic suppression upon KD administration, the liver and kidney showed different responses to gluconeogenesis regulation. While the KD reduced the expression of G6Pase in the liver, suggesting an impairment of hepatic glucose production, expression of kidney G6Pase was maintained (Figure 5). As the kidney is a gluconeogenic organ that can account for up to 15–40% of endogenous glucose production in humans [43,44], we speculate that the kidney may be the major gluconeogenic organ during a ketogenic diet, and, as hepatic G6Pase is decreased under ketosis, the kidney’s gluconeogenic function may secure glucose production. This is consistent with previous studies demonstrating that the kidney can markedly increase gluconeogenic fluxes, particularly from substrates such as glutamine and lactate, under conditions of carbohydrate restriction [3].

Moreover, the regulation by AMPK observed may also contribute to the hepatic repression of G6Pase, as AMPK signaling interferes with gluconeogenic transcriptional regulators such as PGC-1α and FOXO1 [45], while the kidney may be less sensitive to this suppressive axis in the ketogenic state.

Another aspect of our work concerns the impact of ketogenic nutrition on hepatic lipid accumulation. While mice switched from a high-fat diet to a standard chow diet showed a significant regression of steatosis, animals switched to a ketogenic diet retained a considerable amount of hepatic fat, despite other metabolic improvements such as weight loss, fat mass reduction, and normalized glycemia. This finding suggests that hepatic lipid stores established during prolonged exposure to an obesogenic diet display a remarkable resistance to clearance under ketogenic conditions.

The persistence of metabolic dysfunction-associated steatotic liver disease (MASLD) represents a critical problem of obesity and metabolic syndrome [46]. A short-term KD regimen in patients with obesity is recognized as a possible strategy to induce weight loss, particularly within the first few weeks of implementation [47]. While short-term studies in mice (2 weeks) provided hints that KD ameliorates MASLD [48], long-term KD administration (>35 weeks) has been shown to exacerbate fatty liver disease [36]. Our data support that the ketogenic diet, although effective for body weight reduction and glycemic control, may be less efficient in restoring hepatic lipids than standard balanced diets following a previously administered high-fat diet. Indeed, the analysis of the short-term dietary intervention we made offers an additional mechanistic understanding of the contrasting metabolic pathways triggered by carbohydrate restriction (KD) compared to carbohydrate excess (20% saccharose supplementation, Figure 5). In our 7-day study, animals receiving the KD showed a reduction in glycemic levels and displayed ketonemia, confirming the rapid induction of nutritional ketosis. These findings reflect the classical metabolic shift from glucose dependence to fatty acid β-oxidation and ketone body production that occurs when carbohydrate availability is drastically reduced [3,31]. In contrast, the saccharose-supplemented group maintained higher glycemic concentrations. In spite of these metabolic differences, total body weight and fat mass remained unchanged across all groups after one week, indicating that the intervention period was too short to elicit significant changes. However, QMR analysis revealed a reduction in lean mass in both saccharose- and KD-fed animals, with the loss being more pronounced in the ketogenic group. Hence, a reduction in lean mass under either KD or saccharose supplementation may reflect catabolic adaptations to metabolic stress. Additionally, we observed an increase in hepatic (but not kidney) triglycerides after one week of KD, which may be interpreted as the result of a temporary imbalance between lipid synthesis, secretion, and utilization.

Mice exposed for one week to 20% saccharose supplementation displayed, in both the liver and kidney, enhanced lipogenesis, reflected in increased ACC and FASN protein and gene expression, confirming that excess sugar intake promotes anabolic lipid deposition and contributes to steatosis. Furthermore, in this group, we observed reduced levels of Perlipin-2 (*Plin2*), a lipolytic gene (Figure 7B). In contrast, ketogenic feeding induced a profound repression of lipogenic pathways and simultaneously triggered ketogenesis, as demonstrated by gene and protein upregulation of HMGCS2 in both the liver and kidney. In our study, kidney HMGCS2 expression was upregulated after just one week of ketogenic diet, indicating that the enzymatic system required for ketone body synthesis is rapidly activated in response to carbohydrate restriction. As this observation occurred in both nutritional protocols, we conclude that ketogenic responses occur independently of the age and sex of the animals. Notably, the pronounced induction of HMGCS2 observed in the kidney in both models suggests that renal tissues may play an active role in ketogenesis. This observation is consistent with previous evidence that the kidney possesses significant ketogenic potential, particularly under metabolic stress or prolonged fasting conditions [3,18]. Despite the insights provided by this study, several limitations shall be acknowledged. First, the experiments were conducted exclusively in a mouse model, using both sexes in the second protocol (Figure 5), but only male mice in the first protocol (Figure 1). Obviously, the mouse model limits the direct extrapolation of the findings to human physiology. Metabolic responses to ketogenic or sucrose-rich diets may differ substantially between species. Second, the study relied on relatively small sample sizes. Third, the duration of the dietary interventions may not fully capture the long-term metabolic adaptations associated with sustained ketogenic nutrition—especially if benchmarked to the human lifespan. Additionally, the study focused primarily on molecular and biochemical markers of lipid and glucose metabolism, without incorporating other metabolic parameters such as insulin sensitivity, circulating lipid profiles, inflammatory markers, and exploration of all the enzymes involved in the lipogenic (e.g., CPT1A, ACOX1, PPARα) and gluconeogenic (PEPCK) pathways.

## 5. Conclusions and Future Perspectives

In summary, during ketosis, we observed a decreased expression of lipogenic enzymatic machinery both in the liver and kidney, leading to a shift in the body’s metabolic state towards increased fat oxidation and ketone body production. This response was robust, as it occurred both in male and female mice, at different ages, and with different durations of the KD. Provision of a KD was associated with reduced hepatic G6Pase protein expression, but retained G6Pase expression in the kidney, suggesting that under a ketogenic diet, the kidney may serve as a gluconeogenic organ. Finally, a short-term administration of a KD versus a carbohydrate-enriched diet (via saccharose supplementation) underlined in both nutritional patterns a short-term significant loss of body lean mass, suggesting that diets extremely enriched in either carbohydrates or fat may, in the end, provoke deleterious effects, as also suggested by epidemiological observations in large cohorts [49,50]. Future research should explore how these dietary interventions influence inter-organ metabolic communication, particularly between the liver, kidney, adipose tissue, and skeletal muscle. Ultimately, translating these findings into well-designed human studies will be essential to determine the clinical relevance of ketogenic dietary strategies and their potential metabolic consequences.

## Data Availability

All datasets used and analyzed are available from the corresponding author upon request.

## Supplementary Materials

The following supporting information can be downloaded at: https://www.mdpi.com/article/10.3390/nu18071141/s1, Table S1: Macronutrient composition of diets used in this study, Table S2: qPCR primers used in this study.
