# Supplementary material for: Divergent Liver and Kidney Metabolic Responses to Ketogenic, High-Fat, and Sucrose-Enriched Diets in Mice
Source: Nutrients. 2026 Apr 1;18(7):1141. doi: 10.3390/nu18071141 (PMC13074964; doi:10.3390/nu18071141)
Supplement: Supplementary file 1 [file nutrients-18-01141-s001.zip › nutrients-4202187-supplementary.pdf]

Supplementary Table S1: Macronutrient composition of diets used in this study

Diet	High Fat Diet, HFD	Ketogenic diet, KD	Chow diet, CD
**Provided by (Company Name)**	Envigo/harlan Taklad	SSNIFF Spezialdiäten, Soest, Germany	LASQCdiet®,Rod16, LASvendi, Soest, Germany
**Catalog Number**	MD.99249	EF R/M with 80% Fat	D.Rod16.RL7
**Gross energy content (MJ/kg)**	23.02	34.9	15.9
**% of fat by weight**	36.1	94	4.3
**% of proteins by weight**	20.0	8.0	16.9
**% of carbohydrates by weight**	35.1	< 1% (Sugar content is 0,7 % w/w in this diet)	51.2

Supplementary Table S2: qPCR primers used in this study

Gene name	Primers (S, sense ; AS, antisense)	Gene symbol	Ref seq number	Amplicon size, base pairs	Annealing temperature
Acetyl-Coenzyme A acetyltransferase	S :AGACATTGCCATGTGGGAAG<br>AS:CAAATACTAGCCAGACCGAAC	*Acc*	NM_144784.3	204	60 °C
Beta actin	S :AGCCATGTACGTAGCCATCC<br>AS:TCCCTCTCAGCTGTGCTGGTGAA	*Actb*	NM_007393	231	60 °C
Fibroblast growth factor 21	S :GGTACCTCTACACAGATGAC<br>AS:AAGTGAGGCGATCCATAGAG	*Fgf21*	NM_020013	208	60° C
3-hydroxy-3-methylglutaryl-Coenzyme A synthase 2	S :GCTGCCAACTGGATGGAG<br>AS:GTCGTACGCGTTCTCCATGT	*Hmgcs2*	NM_008256	195	60° C
Perilipin	S : CCTATTCTGAACCAGCCAAC<br>AS: CTGCTCCTTTGGTCTTATCC	*Plin2*	NM_007408	151	60° C
Fatty acid synthase	S : CTGTGCTTGCAGCTTACTGG<br>AS: ACGGTGTCCTCAGAGTTGTG	*Fasn*	NM_07988	151	60° C
